# Fulminant Hepatic Failure in a Patient with Crohn's Disease on Infliximab Possibly Related to Reactivation of Herpes Simplex Virus 2 Infection

**DOI:** 10.1155/2016/2132056

**Published:** 2016-10-12

**Authors:** Gary Golds, Lawrence Worobetz

**Affiliations:** ^1^Department of Medicine, University of Saskatchewan, 103 Hospital Drive, Saskatoon, SK, Canada S7N 0W8; ^2^Division of Gastroenterology, Department of Medicine, University of Saskatchewan, 103 Hospital Drive, Saskatoon, SK, Canada S7N 0W8

## Abstract

HSV hepatitis is a rare but often fatal cause of liver failure which tends to affect immunocompromised individuals. Early treatment with Acyclovir has been shown to reduce mortality in HSV hepatitis making recognition of the condition critically important. Here, we present a case of HSV hepatitis in a young woman with Crohn's disease on Prednisone, Azathioprine, and Infliximab. We discuss the clinical presentation of HSV hepatitis as well as the possible causes of hepatitis in a patient on these medications. This case helps demonstrate the importance of early clinical suspicion for HSV in undifferentiated fulminate liver failure. It is also the first reported case of HSV hepatitis in a patient on Infliximab, raising the possibility of HSV reactivation in patients on Infliximab.

## 1. Introduction

Herpes simplex virus (HSV) is a rare but often fatal cause of acute liver failure which generally affects immunosuppressed individuals and pregnant women in the third trimester [1]. Mortality rates for HSV hepatitis are high, but early intervention with Acyclovir appears to greatly reduce morality. As such, early clinical recognition of HSV hepatitis can be potentially life-saving. Many patients with inflammatory bowel disease (IBD) utilize immunomodulators and biologic therapies to help control their disease. It has long been known that these agents, particularly the tumor necrosis factor-alpha (TNF*α*) inhibitors such as Infliximab, put patients at increased risk for opportunistic infections and reactivation of latent infections such as tuberculosis. Here we report a case of HSV-2 reactivation causing fulminant liver failure in a patient with IBD recently started on Infliximab.

## 2. Case Presentation

A 33-year-old female with Crohn's disease presented with a two-day history of subjective fevers and chills and right upper quadrant pain. Her past medical history included endometriosis, cholecystectomy, previous liver abscess, and Crohn's disease. Her medications were venlafaxine, norgestimate/ethinylestradiol contraceptive pill, Azathioprine 100 mg daily, Infliximab, and Prednisone. The Infliximab was started 5 months prior to presentation at a dose of 300 mg every four weeks with the most recent dose 9 days prior to symptom onset. Prednisone was started two weeks priorly for Crohn's flare and was being tapered at 5 mg per week with a dose of 40 mg daily at the time of admission. The patient had no known drug allergies and no family history of autoimmune disease or liver disease.

The patient presented to her local hospital on her second day of symptoms. Her temperature was 38.9°C for which she received a dose of acetaminophen. Initial investigations revealed elevated ALT and AST of 2210 U/L and 2935 U/L, respectively. She was then transferred to the care of the Gastroenterology Team at Royal University Hospital for further assessment.

Regarding her history, she denied any medication changes, over the counter medication use, or intentional ingestions. There were no new food exposures, sick contacts, intravenous drug use, new sexual partners, or recent alcohol use. She denied pruritus, jaundice, change in mental status, nausea or vomiting, rashes, inflamed joints, or eye changes. Her bowel movements were once daily with no blood or mucous or melena and she had no abdominal cramping. She did report a two-day history of white oral plaques and odynophagia without dysphagia. She had travelled to the Dominican Republic three months priorly and felt well during and after the trip.

On exam, vital signs were within normal limits including a temperature of 36.5°C. Cardiovascular and respiratory exam were unremarkable except for signs of mild dehydration. Abdomen was nonprotuberant with bowel sounds present. There was tenderness to palpation in the right upper quadrant with voluntary guarding. The liver edge was palpable just below the costal margin and was smooth and nontender. Traube's space was resonant suggesting no splenomegaly. There were no stigmata of chronic liver disease, and the patient did not exhibit asterixis. In the mouth there were multiple small nonvesicular white plaques with surrounding erythema.

**Figure 1:**
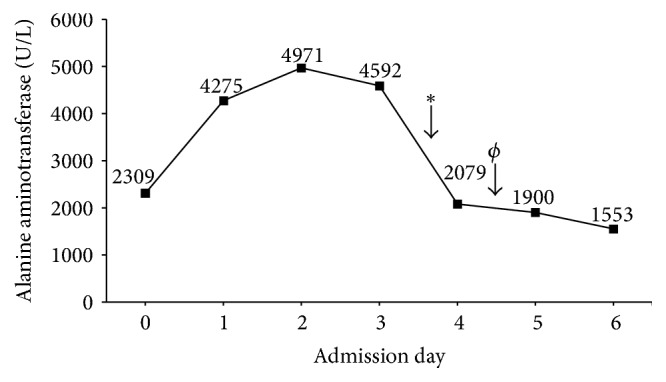
Alanine aminotransferase (ALT) levels during hospital admission in relation to medical therapy. Solumedrol initiation is denoted by *∗* while initiation of Acyclovir is indicated by *ϕ*.

Laboratory investigations showed ALT 2309 U/L, AST 3481 U/L, AP 80 U/L, GGT 50 U/L, Total Bilirubin 6 *μ*mol/L, Albumin 28 g/L, and INR 1.3. White blood cells were normal at 5.96 × 10*e*9/L with a normal differential except for mildly diminished monocyte count of 0.18 × 10*e*9/L. Hemoglobin was normal but platelets were low at 111 × 10*e*9/L. Serum acetaminophen level was positive at 20 *μ*mol/L, but based on the modified Rumack-Matthew nomogram the patient did not meet criteria for N-acetylcysteine administration. The patient was admitted to hospital for further investigations and was started on nystatin oral suspension for suspected oral candidiasis. On postadmission day (PAD) 1 the patient's ALT and AST continued to rise as did her INR and bilirubin (Figure 1). Abdominal ultrasound with Doppler was normal with no evidence of Budd-Chiari syndrome. Infectious workups for EBV, CMV, hepatitis C, and hepatitis B were all negative, but hepatitis A IgG was positive with IgM pending. Investigations for autoimmune hepatitis and Wilson's disease were also still pending. Based upon these initial results it was thought that the patient had either an acute viral or autoimmune hepatitis.

On PAD 2 the patient developed tachycardia and her platelets continued to decline. There was laboratory evidence of disseminated intravascular coagulation (DIC) with decreased fibrinogen, elevated D-dimer, haptoglobin, and LDH. Hematology was consulted and they confirmed the diagnosis of DIC. The patient was started on a two-day course of intravenous immune globulin and given fresh frozen plasma and cryoprecipitate. Platelet transfusion was recommended but withheld due to lack of Rh− platelets being available. Hepatitis A IgM also came back negative and the patient was started on Solumedrol 40 mg IV every 8 hours for presumed autoimmune hepatitis. Azathioprine was also stopped given the possibility for drug induced hepatotoxicity.

On PAD 3 the ANA titre came back positive at 1 : 160 which further suggested autoimmune hepatitis, and as such IV steroids were continued. The liver transplant team was notified of the patient and plans for liver biopsy were made, but due to ongoing severe coagulopathy and clinically significant bleeding, the biopsy was delayed for fear of significant postprocedure bleeding. On PAD 4 the patient developed spontaneous hematemesis prompting IV pantoprazole infusion, ICU admission, and emergent gastroscopy. This demonstrated a normal stomach and duodenum but diffuse esophagitis with mucosal sloughing suspicious for an infectious process. Biopsies of the esophagus were taken and the infectious disease team was consulted. Subsequently, multiple erosive lesions on the vulva which appeared herpetiform in nature were discovered. The patient was then started on Acyclovir 300 mg IV every 8 hours for a suspected disseminated HSV infection and the Solumedrol was stopped. Due to the patient's travel history, investigations for potentially liver toxic pathogens endemic to the Dominican Republic (hepatitis E, yellow fever virus, dengue virus, chikungunya virus, and* Leptospira*) were also done. These all eventually came back negative.

The next day, the patient's clinical status further declined with decreasing level of consciousness necessitating intubation. Her change in mental status was thought to be from hepatic encephalopathy and she was given lactulose via nasogastric tube. She was started on meropenem in case of concomitant bacteria infection. The patient then became anuric and went into respiratory failure and after aggressive resuscitation attempts the patient passed away. An autopsy was declined by the patient's family.

The results of the esophageal biopsy came back positive for fungal hyphae as well as HSV-2. Swabs from the vulvar lesions were positive for HSV-2 and serum PCR was also positive for HSV-2 but negative for varicella. Given this clinical picture, the patient was determined to have died from fulminant HSV-2 hepatitis with concomitant DIC and multiorgan failure. The fact that the patient had HSV-2 IgG suggests this was likely a reactivation of a previous HSV-2 infection.

## 3. Discussion

HSV hepatitis is a rare but often fatal cause of acute liver failure. It likely accounts for less than 1% of all cases of acute liver failure [2], but given its mortality rates of up to 74% [1], it is an important diagnosis to consider in any patient presenting with acute liver failure of unknown origin. HSV hepatitis primarily affects immunosuppressed individuals and pregnant females in their third trimester [1, 3]; however there have been many reported cases of immunocompetent individuals developing HSV hepatitis [4–11]. Patients with HSV hepatitis most commonly present with fever, thrombocytopenia, coagulopathy, and encephalopathy [1]. Cutaneous manifestations of the virus are present in less than half of all cases [1], and most diagnoses are made on autopsy [1]. Even with treatment using Acyclovir, mortality rates of HSV hepatitis are still as high as 51% [1]. Additionally, there is evidence that a delay in administration of Acyclovir is associated with increased mortality rates [12]. The significantly reduced mortality of HSV hepatitis with Acyclovir treatment, as well as its relatively safe drug profile [13, 14], has prompted some clinicians to recommend empiric Acyclovir therapy in any patient presenting with hepatitis who is at risk of HSV infection [15, 16].

The clinical features in our case report are very similar to what has been described for HSV hepatitis in the literature. Given these similarities in presentation, the positive serum PCR, and esophageal biopsy for HSV-2 and previous HSV IgG, the most likely cause of hepatitis in our case is HSV-2 reactivation. Other potential causes of hepatitis to consider in our patient include autoimmune or drug induced causes. In our case, while the patient's serum ANA titre was significant at 1 : 160, the lack of clinical improvement with pulse steroids as well as negative antismooth muscle antibody assay and nonelevated IgG makes autoimmune hepatitis unlikely in our patient. Our patient was on both Infliximab and Azathioprine, two medications known to cause drug induced liver injury. In the case of Azathioprine, drug induced hepatotoxicity can occur over a broad range of time frame and is generally associated with drug initiation or dose changes [17]. In our case, however, there had been no recent changes in Azathioprine and the drug was also discontinued without an improvement in the patient's condition. Therefore, it is unlikely that Azathioprine was the cause of our patient's fulminant liver failure. The anti-TNF*α* agents, primarily Infliximab, have also been reported to cause drug induced liver injury [18]. However, most of these cases only had mild elevation of liver enzymes, occurring months after initiation of the anti-TNF*α* agent [18, 19]. Our patient on the other hand had dramatic elevations in her liver enzymes. Interestingly there have been case reports of ANA positive hepatitis following Infliximab infusions which suggest the possible induction of autoimmune hepatitis by Infliximab [20]. However, given the lack of ANA titres prior to Infliximab administration in many of the patients, it is hard to determine whether development of autoimmune hepatitis was associated with Infliximab administration or merely coincidental. Similarly in our case, our patient had a positive ANA titre with an unknown titre prior to hospitalization.

While HSV hepatitis has not previously been reported in a patient on Infliximab, it is well known that the use of TNF*α* inhibitors can lead to reactivation of latent infections [21]. In terms of viral infections, hepatitis B (HBV) and hepatitis C (HCV) are the best studied in patients on TNF*α* inhibitors [22]. Overall, HBV reactivation is much more common [23, 24] and this is thought to be due to the required involvement of TNF*α* in the host immune response to HBV infection [25, 26]. Similarly, the role of TNF*α* in host defense mechanisms against the herpes simplex virus is thought to be important and likely involves macrophage activation and immune cell signalling [27]. Mouse models have demonstrated that TNF*α* depletion leads to rapid reactivation of HSV-1 infections and TNF*α* knockout mice are more susceptible to primary HSV-1 infection and had higher subsequent rates of mortality [28]. Following from this, it would not be unexpected for patients on TNF*α* inhibitors to have increased rates of viral herpes infections. Indeed, there are a large number of reports of herpes zoster infections in patients on Infliximab as well as case reports of HSV-1 encephalitis, necrotizing tonsillitis, and diffuse cutaneous infection in patients treated with Infliximab [29–32]. Therefore, it would not be unreasonable to believe that HSV reactivation could cause viral hepatitis in a patient on a TNF*α* inhibitor.

In conclusion, we have presented a case of acute hepatitis from HSV-2 reactivation leading to thrombocytopenia, coagulopathy, multiorgan failure, and death despite IV Acyclovir treatment. The patient had recently started taking Infliximab and this is the first case to our knowledge of HSV hepatitis in a patient on this medication [1, 21, 33]. This case raises the possibility of Infliximab causing HSV-2 reactivation in patients leading to hepatitis. It also further emphasizes the need to have a high clinical suspicion for HSV hepatitis in any patient presenting with acute hepatitis who is immunosuppressed as early Acyclovir administration has been demonstrated to significantly reduce mortality [1, 12]. Due to this reduced mortality, the challenge of diagnosing HSV hepatitis, and the relatively safe drug profile of Acyclovir [16], we recommend that any patient who is immunosuppressed and presenting with hepatitis of unknown origin should be started on empiric Acyclovir therapy.
